# Epidemiological analysis of acute poisoning cases based on Poison control center data from 2021 to 2024

**DOI:** 10.1080/07853890.2026.2696600

**Published:** 2026-07-05

**Authors:** Qing Tang, Jiaqi Xu, Xin Luo, Hao Wang, Shuxin Hua, Yanfen Chai, Lijun Wang

**Affiliations:** Emergency Medicine Department, Tianjin Medical University General Hospital, Tianjin, China

**Keywords:** Acute poisoning, emergency treatment, epidemiology, poisoning center

## Abstract

**Background:**

To determine the circumstances of acute poisoning cases at the Poison Control Center (2021–2024) and analyze their risk factors.

**Methods:**

We retrospectively analyzed the demographic characteristics, clinical features, and prognosis of patients with acute poisoning at the Poison Control Center.

**Results:**

This study included 10,402 cases comprising drug poisoning (5,074), alcohol poisoning (2,316), carbon monoxide poisoning (1,803), pesticide poisoning (882), and chemical poisoning (327). The age group with the highest incidence of poisoning was 21–40 years old (37.94%, *p* < 0.01). Women have a higher proportion of drug poisoning (72.59%, *p* < 0.01), while men have a higher proportion of alcohol poisoning (74.31%, *p* < 01). Patients with pesticide poisoning were predominantly educated to the middle school level or below (73.02%, *p* < 0.01), whereas those with alcohol poisoning were mainly educated to the high school level or above (73.88%, *p* < 0.01). Carbon monoxide and pesticide poisoning occur primarily in rural areas. Poisoning incidents at home accounted for 74.32%, alcohol poisoning frequently occurred in entertainment venues (60.58%, *p* < 0.01). Carbon monoxide poisoning is more common in January and December, whereas drug poisoning has a higher incidence throughout the year. Carbon monoxide, chemical and alcohol poisoning were predominantly accidental, whereas drug and pesticide poisoning were mainly intentional. Gastric lavage dominated pestic ide poisoning; Antidotes prevailed in alcohol poisoning.The overall mortality rate was 1.56 %, highest for pesticide poisoning (8.28%, *p* < 0.01).

**Conclusion:**

Different types of poisoning have distinct sociodemographic characteristics, that should be considered when developing prevention and treatment policies. Personalized treatment plans should be tailored to different poisonings.

## 1. Introduction

Acute poisoning has a high incidence and mortality rate, and its incidence is increasing in many countries and regions[1,2]. The Third National Retrospective Survey on Mortality released in China during 2008 revealed that: poisoning and injuries are the fifth leading causes of death among residents, accounting for 10.7% of the total mortality rate [3]. Since 2010, both the incidence rate and the absolute number of patients with acute poisoning in China have shown an upward trend [4]. On a national and global scale, acute poisoning is a significant public health challenge in China [5], requiring urgent and significant attention.

The characteristics of acute poisoning vary by geographical region, season, and sociodemographic factors such as age [6,7]. Therefore, applying epidemiological methods to acute poisoning problems is the best way to understand the characteristics of acute poisoning [8]. Through epidemiological research, it is possible to gain a better understanding of the epidemiological characteristics of acute poisoning. This will enable the formulation of regional and targeted strategies for poisoning prevention, which can be continuously adjusted in response to societal development [9].

Given the significant variation in the prevalence and types of acute poisoning across regions, it is necessary to research acute poisoning incidents on a regional basis [1]. Utilizing hospital data for epidemiological analysis can help identify pressing health issues, evaluate treatment outcomes, and identify high-risk populations, thereby providing a reference for policy decision-making [10].

However, there is a relative lack of epidemiological research on acute poisoning in China, and due to the wide range of poisoning areas, large populations, and different poisoning patterns in different regions, it is necessary to develop acute poisoning prevention policies based on the epidemiological characteristics of acute poisoning in different regions. This study aimed to conduct a descriptive analysis of patients with acute poisoning from a poisoning control center in China to determine the clinical patterns, types of poisoning, and related factors, thereby providing a theoretical foundation for the development of acute poisoning prevention and control policies.

## 2. Materials and methods

### 2.1. Participants

Patient data were sourced from the Poison Control Center in China, which covered individuals treated at 29 hospitals between 2021 and 2024. In total, 10,676 cases were reported. To prevent statistical bias, 202 patients with mixed poisoning were excluded. Additionally, because of the low number of food poisoning cases (39 cases) and animal and plant poisoning cases (33 cases), which lacked representativeness, these cases were excluded. Consequently, the final analysis included 10,402 valid cases, all of which involved Single type of toxic incidents. This research database has undergone strict quality control by professional personnel, confirming that all included cases have integrity at the core data points.

### 2.2. Research methods

This was a retrospective study. Data on patients with poisoning were extracted from the Poison Control Center’s database and independently organized by two doctors, and any disputed data were verified by reporting personnel. Statistical information covered various aspects of acute poisoning, including sex, age, occupation, residential area, cause of poisoning, time of poisoning, type of poisoning, diagnosis, treatment measures, medical conditions, and prognosis. Age was categorized into five groups: ≤20 years, 21–40 years, 41–60 years, 61–80 years, and ≥81 years. Occupation was categorized based on whether the individual had a fixed work into employment, unemployment, students, and retired individuals. Residential areas were classified as rural or urban, based on the registered residence. In the study of population in Chinese society, this is a widely accepted and applied basic classification method. The cause of poisoning was categorized as accidental or intentional based on whether the patient actively ingested a toxic substance. The patient’s condition was graded according to the PSS guidelines. level 0: No symptoms or signs of poisoning. level 1: Transient, self-limiting symptoms or signs. level 2: Prominent, persistent symptoms or signs; organ dysfunction present. level 3: Severe, life-threatening symptoms or signs; severe organ dysfunction present. Level 4: Death.

### 2.3. Statistical methods

Data analyses were conducted using Excel and SPSS v26.0. Categorical data were presented as percentages (%), and group comparisons were performed using the chi-square test. Statistical significance was set less than 0.05.

### 2.4. Ethics statement

This study had adhered to the principles stated in the ‘Declaration of Helsinki’ and the study protocol was approved by the Ethics Committee of Tianjin Medical University General Hospital. Due to the retrospective nature of the study and the anonymity of patients during the study, the Ethics Committee of Tianjin Medical University General Hospital waived the requirement for informed consent from patients. Ethical code:IRB2024-YX-444-01.

## 3. Results

### 3.1. Sociodemographic characteristics of acute poisoning

**Figure 1.**
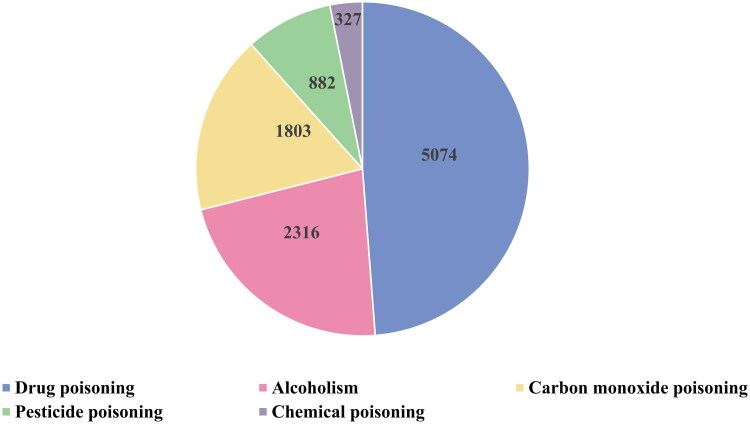
Number of patients with different types of poisoning.

A total of 10,402 patients with single-substance poisoning were included in this study. All poisoning cases were categorized by type as follows: drug poisoning (5,074 cases), alcohol poisoning (2,316 cases), carbon monoxide poisoning (1,803 cases), pesticide poisoning (882 cases), and chemical substance poisoning (327 cases) (Figure 1). Among the patients, 4,643 (44.64%) cases were men and 5,759(55.36%) cases were women, with a men-to-women ratio of 1:1.24. Patient age ranged from 1 to 97 years, with a median age of 38 years. The 21–40 years age group accounted for the highest proportion of patients (37.94%). Furthermore, 40.76% were unemployed, and 57.17% resided in urban areas. 74.32% of poisoning incidents occurred at home (Table 1).

**Table 1.** Sociological factors of acute poisoning.

Classification		Types of poisoning (case, %)					Total	χ^2^	P
		Carbon monoxide poisoning	Pesticide poisoning	Chemical poisoning	Drug poisoning	Alcoholism			
Sex	Woman	953(52.86)	413(46.83)	115(35.17)	3683(72.59)	595(25.69)	5759(55.36)	1518.734	0.000**
	Man	850(47.14)	469(53.17)	212(64.83)	1391(27.41)	1721(74.31)	4643(44.64)		
Age	≤20	119(6.60)	88(9.98)	50(15.29)	1265(24.93)	253(10.92)	1775(17.06)	1108.298	0.000**
	21–40	531(29.45)	298(33.79)	117(35.78)	1731(34.12)	1269(54.79)	3946(37.94)		
	41–60	606(33.61)	275(31.18)	98(29.97)	962(18.96)	642(27.72)	2583(24.83)		
	61–80	452(25.07)	191(21.66)	53(16.21)	858(16.91)	138(5.96)	1692(16.27)		
	≥80	95(5.27)	30(3.40)	9(2.75)	258(5.08)	14(0.60)	406(3.90)		
Education	Below junior high school	1011(56.07)	644(73.02)	174(53.21)	2497(49.21)	605(26.12)	4931(47.40)	1020.326	0.000**
	Above high school	792(43.93)	238(26.98)	153(46.79)	2577(50.79)	1711(73.88)	5471(52.60)		
Occupation	Student	121(6.71)	78(8.84)	53(16.21)	1232(24.28)	311(13.43)	1795(17.26)	1258.102	0.000**
	Unemployed	930(51.58)	658(74.60)	131(40.06)	1741(34.31)	780(33.68)	4240(40.76)		
	Employed	707(39.21)	117(13.27)	115(35.17)	1546(30.47)	1164(50.26)	3649(35.08)		
	Retiree	45(2.50)	29(3.29)	28(8.56)	555(10.94)	61(2.63)	718(6.90)		
Residence	Rural area	1259(69.83)	675(76.53)	129(39.45)	1620(31.93)	772(33.33)	4455(42.83)	1278.97	0.000**
	Urban	544(30.17)	207(23.47)	198(60.55)	3454(68.07)	1544(66.67)	5947(57.17)		
Location	Entertainment venue	193(10.70)	19(2.15)	21(6.42)	25(0.49)	1403(60.58)	1661(15.97)	5481.664	0.000**
	School	7(0.39)	4(0.45)	13(3.98)	164(3.23)	15(0.65)	203(1.95)		
	Home	1399(77.59)	768(87.07)	198(60.55)	4700(92.63)	666(28.76)	7731(74.32)		
	occupational environment	66(3.66)	16(1.81)	55(16.82)	20(0.39)	22(0.95)	179(1.72)		
	Others	138(7.65)	75(8.50)	40(12.23)	165(3.25)	210(9.07)	628(6.04)		

* *p* < 0.05.

** *p* < 0.001.

Significant differences existed in sociodemographic factors between patient groups with different types of acute poisoning (*p* < 0.01). A higher proportion of women were observed among patients with drug poisoning (72.59%) and carbon monoxide poisoning (52.86%), whereas a higher proportion of men were observed among patients with alcohol poisoning (74.31%), pesticide poisoning (53.17%), and chemical substance poisoning (64.83%). The 21–40 age group had the highest number of patients with drug poisoning (34.12%), pesticide poisoning (33.79%), alcohol poisoning (54.79%), and chemical substance poisoning (35.78%). For carbon monoxide poisoning, the highest number of patients (33.61%) was in the 41–60 age group (Table 1).

Regarding educational level, patients with pesticide, carbon monoxide, and chemical substance poisoning were predominantly those below junior high school level, comprising 73.02%, 56.07%, and 53.21%, respectively. In contrast, patients with alcohol and drug poisoning were more likely to have an education above high school, accounting for 73.88% and 50.79%. A higher proportion of unemployed individuals was observed among patients with carbon monoxide poisoning (51.58%), pesticide poisoning (74.60%), chemical substance poisoning (40.06%), and drug poisoning (34.31%). Conversely, among patients with alcohol poisoning, those with stable employment constituted the largest proportion, reaching 50.26% (Table 1).

Regarding the residential distribution, carbon monoxide poisoning and pesticide poisoning primarily occurred in rural areas, accounted for 69.83% and 76.53%. Conversely, patients with chemical substance, drug and alcohol poisoning were predominantly urban residents, accounting for 60.55%, 68.07%, and 66.67%. Alcohol poisoning was most frequent in entertainment venues (60.58%) (Table 1).

### 3.2. Time distribution of acutely poisoned patients

**Figure 2.**
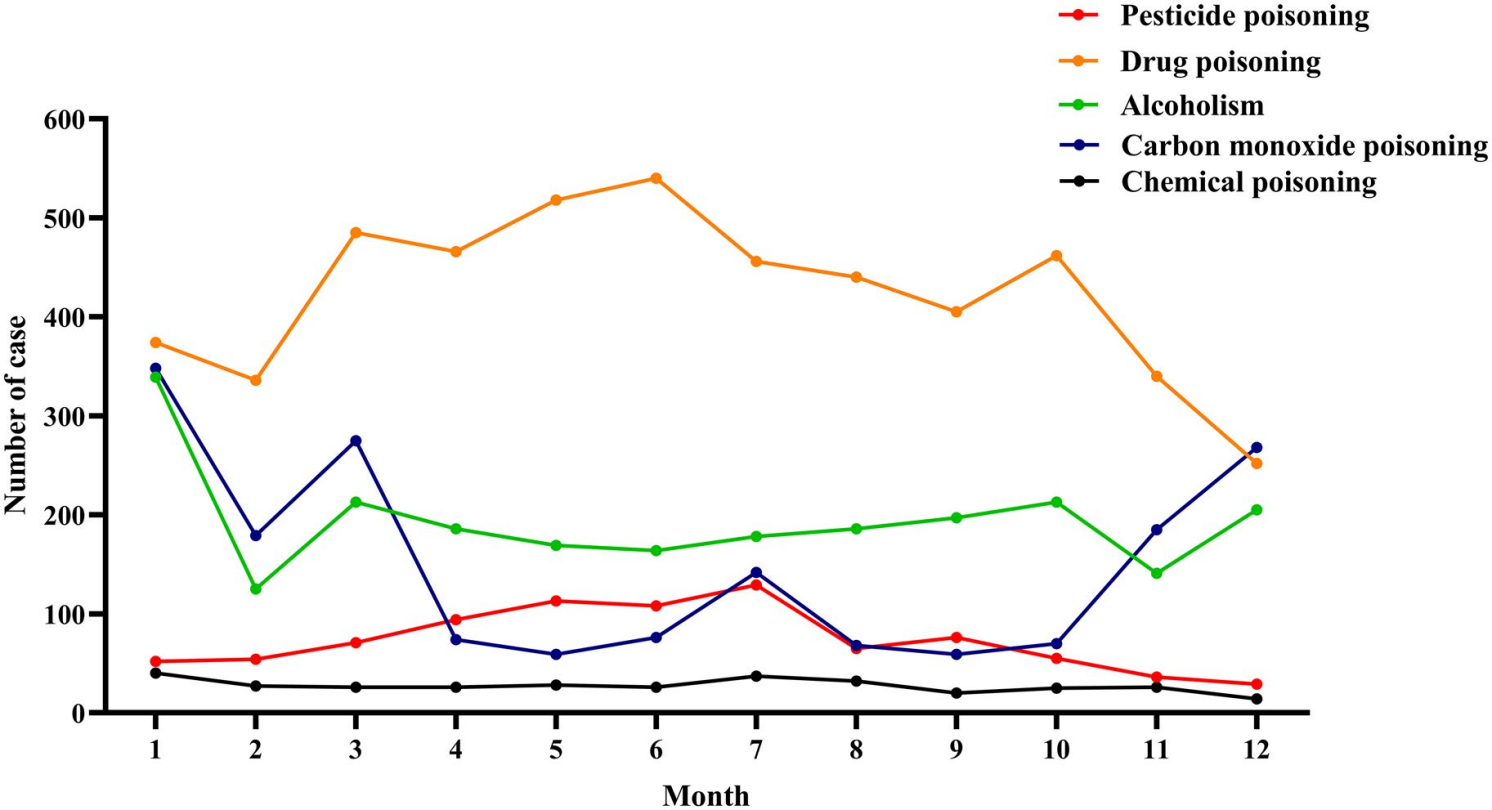
Number of acute poisoning patients in different months (2021–2024).

The types of poisoning vary in different months, with certain types being more common during specific months. Carbon monoxide poisoning is more prevalent in January and December, whereas pesticide poisoning peaks in May and July. Alcohol poisoning was most frequent in January. In contrast, the incidence of drug poisoning was consistently high throughout the year (Figure 2).

### 3.3. Analysis of the causes of acute poisoning in patients

**Figure 3.**
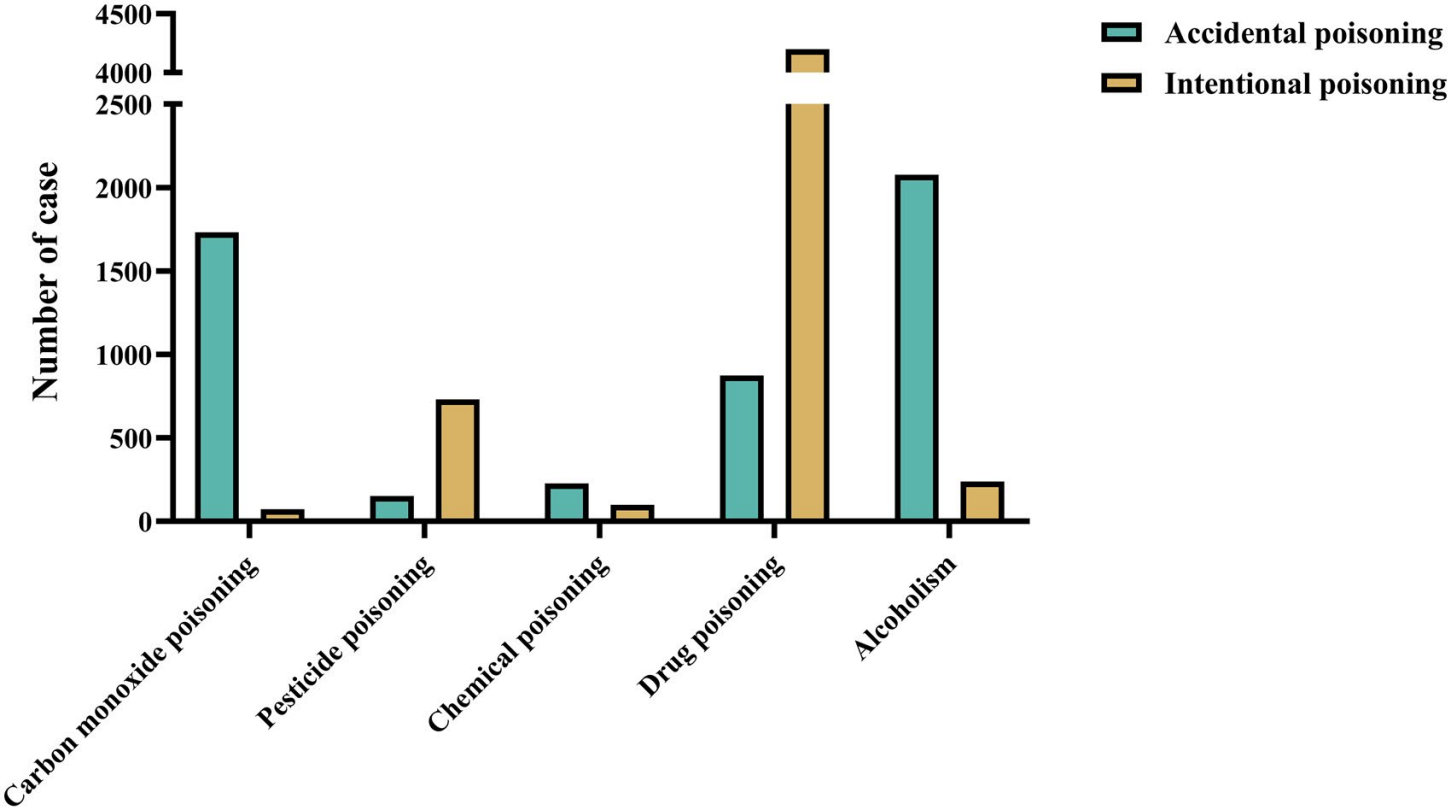
Causes of poisoning in patients with different types of poisoning.

Among the acute poisoning cases, 48.66% were accidental poisoning and 51.34% were intentional poisoning. Further analysis of the different types of poisoning revealed that carbon monoxide, chemical, and alcohol poisonings were predominantly accidental, accounting for 96.01%, 69.72%, and 89.68%. In contrast, drug and pesticide poisoning were primarily intentional, accounting for 82.77% and 82.76% (Figure 3).

### 3.4. Management, clinical course, and prognosis of acute poisoning patients

There are differences in clinical treatment measures for different types of poisoning(*p* < 0.01). The rates of gastric lavage for pesticide and drug poisoning were relatively high at 78.57% and 66.75%. However, the usage rates of vomiting, purgation, activated carbon adsorption and whole-gut irrigation are relatively low for various types of poisoning. However, the application rate of blood purification for pesticide poisoning is relatively high, with 19.39% of the patients with pesticide poisoning using blood purification. The usage rates of Antidotes for alcohol and pesticide poisoning were relatively high at 71.03% and 44.37% (Table 2).

**Table 2.** Treatment measures for acute poisoning.

Treatment		Types of poisoning (case,%)					Total	χ^2^	p
		Carbon monoxide poisoning	Pesticide poisoning	Chemical poisoning	Drug poisoning	Alcoholism			
Gastric lavage	NO	1803(100.00)	189(21.43)	175(53.52)	1687(33.25)	2128(91.88)	5982(57.51)	4146.168	0.000**
	YES	0(0.00)	693(78.57)	152(46.48)	3387(66.75)	188(8.12)	4420(42.49)		
Vomiting	NO	1803(100.00)	775(87.87)	288(88.07)	4425(87.21)	2201(95.03)	9492(91.25)	335.016	0.000**
	YES	0(0.00)	107(12.13)	39(11.93)	649(12.79)	115(4.97)	910(8.75)		
Purgation	NO	1803(100.00)	810(91.84)	315(96.33)	4461(87.92)	2188(94.47)	9577(92.07)	301.526	0.000**
	YES	0(0.00)	72(8.16)	12(3.67)	613(12.08)	128(5.53)	825(7.93)		
Activated carbon adsorption	NO	1803(100.00)	817(92.63)	322(98.47)	4873(96.04)	2311(99.78)	10126(97.35)	213.581	0.000**
	YES	0(0.00)	65(7.37)	5(1.53)	201(3.96)	5(0.22)	276(2.65)		
whole-gut irrigation	NO	1803(100.00)	840(95.24)	324(99.08)	5063(99.78)	2313(99.87)	10343(99.43)	305.083	0.000**
	YES	0(0.00)	42(4.76)	3(0.92)	11(0.22)	3(0.13)	59(0.57)		
Blood purification	NO	1803(100.00)	711(80.61)	315(96.33)	4935(97.26)	2305(99.53)	10069(96.80)	864.624	0.000**
	YES	0(0.00)	171(19.39)	12(3.67)	139(2.74)	11(0.47)	333(3.20)		
Antidotes	NO	1003(55.63)	633(71.77)	292(89.30)	3642(71.78)	671(28.97)	6241(60.00)	1404.453	0.000**
	YES	800(44.37)	249(28.23)	35(10.70)	1432(28.22)	1645(71.03)	4161(40.00)		

* *p* < 0.05.

** *p* < 0.001.

Most patients (61.90%) presented with Level1 severity, and the vast majority (97.58%) did not experience shock. Most patients showed improvement, with an overall mortality rate of 1.56%. However, we also found that severity and mortality rates varied among the different types of poisoning. Pesticide poisoning was the most severe, with the highest proportion of patients (20.87%) presenting with LeveL3 or higher and a higher mortality rate (8.28%) (Table 3).

**Table 3.** Diagnosis, condition, and prognosis of patients with acute poisoning.

Classification		Types of poisoning (case,%)					Total	χ^2^	*P*
		Carbon monoxide poisoning	Pesticide poisoning	Chemical poisoning	Drug poisoning	Alcoholism			
Prognosis	Survival	1801(99.89)	809(91.72)	315(96.33)	5008(98.70)	2307(99.61)	10240(98.44)	316.673	0.000**
	Death	2(0.11)	73(8.28)	12(3.67)	66(1.30)	9(0.39)	162(1.56)		
State of illness(PSS)	Level0	250(13.87)	150(17.01)	96(29.36)	1125(22.17)	331(14.29)	1952(18.77)	1063.295	0.000**
	Level1	1304(72.32)	354(40.14)	154(47.09)	2854(56.25)	1773(76.55)	6439(61.90)		
	Level2	221(12.26)	194(22.00)	58(17.74)	855(16.85)	187(8.07)	1515(14.56)		
	Level3	26(1.44)	111(12.59)	7(2.14)	174(3.43)	16(0.69)	334(3.21)		
	Level4	2(0.11)	73(8.28)	12(3.67)	66(1.30)	9(0.39)	162(1.56)		
Toxic detection	NO	419(23.24)	423(47.96)	228(69.72)	2678(52.78)	2210(95.42)	5958(57.28)	2324.813	0.000**
	YES	1384(76.76)	459(52.04)	99(30.28)	2396(47.22)	106(4.58)	4444(42.72)		
Shock	NO	1796(99.61)	814(92.29)	314(96.02)	4934(97.24)	2292(98.96)	10150(97.58)	160.461	0.000**
	YES	7(0.39)	68(7.71)	13(3.98)	140(2.76)	24(1.04)	252(2.42)		

* *p* < 0.05.

** *p* < 0.001.

## 4. Discussion

In this study, most patients had drug poisoning. Recently, the incidence of drug poisoning has increased. Other studies in China have also shown that drug poisoning accounts for a higher proportion in acute poisoning [11]. In China, the relative ease of access to medications, partly owing to irregularities in prescription regulations, has contributed to acute drug poisoning. To reduce the incidence of acute drug poisoning effectively, it is essential to strengthen drug regulations and decrease medication accessibility.

In the study, significant sex differences were observed among patients with drug, alcohol, and chemical poisoning. Sex plays a significant role in the poisoning [12]. Women are more susceptible to drug poisoning, which may be related to the following reasons. First, women are more likely to suffer from mental health conditions such as mood disorders, anxiety disorders, and sleep disorders [13,14]. They have more opportunities to come into contact with sedatives and psychiatric medications[14]. Secondly, women face higher levels of psychological stress due to work and family pressures, contributing to a relatively high suicide rate among Chinese women [15,16]. However, regarding alcohol poisoning, we found that men significantly outnumbered women, which is associated with males engaging in more social and business engagements. In research on chemical substance poisoning, we observed that men also outnumbered women, a result that contradicts the findings of other studies [17]. This discrepancy may be attributed primarily to cultural differences across countries. In China, males constitute most workers in chemical-exposure industries, resulting in a greater risk of chemical poisoning among this group.

The incidence of poisoning was notably higher in the 21–40 and 41–60 age groups, which is consistent with findings from related studies [1,18]. This could be attributed to the fact that individuals in these age groups are more likely to encounter various toxic substances, thereby increasing their risk of poisoning. Over the past decade, there has been a continuous increase in poisoning incidents among younger populations [19], we must consider this seriously.

There were also significant differences in the education levels of patients with different types of poisoning. A study has found that pesticide poisoning is more likely to occur in populations with lower levels of education [20],which is consistent with our findings. This may be attributed to the fact that lower-educated individuals reside more frequently in rural areas, where they are more likely to contact with pesticides. Additionally, higher education levels are associated with higher blood alcohol concentrations upon hospital admission [21], and approximately two-thirds of college students reported experiencing frequent drinking or even alcohol abuse[22], resulting in a relatively higher level of education among patients with alcohol poisoning.

Occupational factors significantly affect acute poisoning [23]. Our study found that among patients with acute poisoning, the majority did not have a fixed occupation. This elevated risk may be attributable to frequent engagement in high-risk temporary work involving hazardous chemical exposure, compounded by a lack of systematic occupational training and safety protection. For occupations that may accept toxic substances, safety training should be further strengthened

Acute poisoning has significant regional differences [6]. Patients with carbon monoxide poisoning and pesticide poisoning are mostly rural patients. In rural areas, the widespread use of coal and pesticides increases their risk of poisoning. By upgrading cooking facilities in rural areas, reducing carbon monoxide emissions, and promoting the installation of safety equipment such as carbon monoxide alarms, the occurrence of carbon monoxide poisoning incidents can be effectively prevented and reduced [24]. Strengthening pesticide management and standardizing pesticide use in rural areas is necessary to reduce pesticide poisoning incidents.

A considerable proportion of acute poisoning cases occurs in households, whereas alcohol poisoning predominantly occurs in entertainment venues. In entertainment venues, excessive drinking is perceived as a form of recreation and is sometimes encouraged. This cultural atmosphere increases the risk of alcohol poisoning. Alcohol poisoning not only causes serious health damage to the patient [25], but also has a significant impact on public safety through alcohol-related violent behavior [26]. Posting promotional posters at entertainment venues to promote healthy drinking culture can effectively reduce alcohol poisoning.

Acute poisoning showed a clear seasonal trend [27]. During winter, owing to the increased use of coal for heating, January and December become the peak periods for carbon monoxide poisoning. Additionally, January marks the end of the year, when various gatherings are frequent, leading to an elevated risk of alcohol poisoning. Therefore, it is important to implement preventive measures against different types of poisoning during different periods.

Suicide is a serious problem worldwide [28], and this study found that over half of the patients were intentionally poisoned. Implementing the necessary psychological interventions for specific populations while restricting their access to means of suicide, such as strengthening the management of medications and pesticides, holds significant importance in preventing suicidal behaviors. However, carbon monoxide, chemical, and alcohol poisoning are primarily accidental. Effective occupational protective measures can significantly reduce the incidence of such incidents.

Prompt implementation of measures to eliminate toxins, including gastric lavage, activated charcoal adsorption, vomiting, purgation, and whole-gut irrigation, while expediting their excretion, forms the foundation of treatment for acute drug poisoning [29]. However, there is currently insufficient evidence to improve patient prognosis through these measures and the complications they cause cannot be ignored, leading to controversy regarding their clinical applications [30]. Further research is needed to clarify its indications and effects. Notably, some patients with alcohol poisoning also received gastric lavage treatment, although it is currently not recommended to routinely perform gastric lavage in patients with alcohol poisoning [31]. This finding suggests that there may be excessive or inappropriate use of gastric lavage in clinical practice. It is necessary to strengthen the training of clinical doctors and promote guidelines to ensure the rationality and scientific validity of treatment measures.

Management of patients with acute poisoning requires the timely use of antidotes to minimize sustained toxicity and mortality [32]. In this study, less than one-fifth of patients used antidotes, and the proportion of patients with chemical, drug, and pesticide poisoning treated with antidotes was even lower. Because antidotes did not cover all poisons [33]. Hospitals should store necessary antidotes and develop new and efficient antidotes to improve the treatment rate of acute poisoning.

The mortality rate of pesticide poisoning in this study was 8.28%, indicating that pesticide poisoning is more severe. It has always been a serious social problem, but it can be prevented [34]. Reducing the use of highly hazardous pesticides and the concentrations of pesticides in formulations can effectively reduce pesticide poisoning [35].

This study mainly emphasizes the important role of sociodemographic characteristics in the prevention and treatment of different types of acute poisoning. By understanding these characteristics, professionals can more accurately identify high-risk populations and develop targeted intervention strategies for them, thereby reducing the occurrence of acute poisoning.

The data we studied only came from one poison control center. The data only represents cases reported to the center and may omit unreported or mild cases, resulting in research bias. Additionally, this data is only representative of the region. Future research should include multiple centers to obtain data from a wider and more representative population, to improve the reliability and application value of research conclusions, and establish a nationwide acute poisoning reporting system.

## 5. Conclusion

Drug poisoning is the most common type of poisoning and pesticide poisoning is the main cause of death. The social and demographic characteristics of different types of poisoning vary, and these factors must be considered when implementing treatment and prevention policies. The treatment of acute poisoning requires further standardization.

## Supplementary Material

STROBE _checklist.docx

## Data Availability

Due to patient privacy concerns, the data supporting the results of this study are not publicly available but can be obtained from the corresponding author upon reasonable request. The data are stored in a controlled access data repository at Tianjin Medical University General Hospital. This database is owned by the Poison Control Center, and the corresponding author of this article has full access to it. We can directly export the data and conduct searches. Corresponding author email: wanglijun211022@tmu.edu.cn
